# Comparative gut microbiome research through the lens of ecology: theoretical considerations and best practices

**DOI:** 10.1111/brv.13161

**Published:** 2024-11-12

**Authors:** Samuel Degregori, Xiaolin Wang, Akhil Kommala, Noah Schulhof, Sadaf Moradi, Allison MacDonald, Kaitlin Eblen, Sophia Jukovich, Emma Smith, Emily Kelleher, Kota Suzuki, Zoey Hall, Rob Knight, Katherine Ryan Amato

**Affiliations:** ^1^ Department of Anthropology Northwestern University 1810 Hinman Avenue Evanston IL 60208 USA; ^2^ Department of Ecology and Evolutionary Biology University of California 621 Young Drive South Los Angeles CA 90095 USA; ^3^ Department of Pediatrics University of California San Diego La Jolla CA 92093 USA

**Keywords:** gut microbiome, comparative, ecology, host–microbe interactions, evolution

## Abstract

Comparative approaches in animal gut microbiome research have revealed patterns of phylosymbiosis, dietary and physiological convergences, and environment–host interactions. However, most large‐scale comparative studies, especially those that are highly cited, have focused on mammals, and efforts to integrate comparative approaches with existing ecological frameworks are lacking. While mammals serve as useful model organisms, developing generalised principles of how animal gut microbiomes are shaped and how these microbiomes interact bidirectionally with host ecology and evolution requires a more complete sampling of the animal kingdom. Here, we provide an overview of what past comparative studies have taught us about the gut microbiome, and how community ecology theory may help resolve certain contradictions in comparative gut microbiome research. We explore whether certain hypotheses are supported across clades, and how the disproportionate focus on mammals has introduced potential bias into gut microbiome theory. We then introduce a methodological solution by which public gut microbiome data of understudied hosts can be compiled and analysed in a comparative context. Our aggregation and analysis of 179 studies shows that generating data sets with rich host diversity is possible with public data and that key gut microbes associated with mammals are widespread across the animal kingdom. We also show the effects that sample size and taxonomic rank have on comparative gut microbiome studies and that results of multivariate analyses can vary significantly with these two parameters. While challenges remain in developing a universal model of the animal gut microbiome, we show that existing ecological frameworks can help bring us one step closer to integrating the gut microbiome into animal ecology and evolution.

## I. INTRODUCTION

Gut microbiomes are critical components of animal physiology and survival (Kinross, Darzi & Nicholson, 2011; Gibson *et al*., 2019; Worsley *et al*., 2021; Comizzoli *et al*., 2021). As a result, comparative gut microbiome studies are integral to understanding animal ecology and evolution. The beginning of comparative gut microbiome approaches is best highlighted by Ley *et al*. (2008) which leveraged 16S high‐throughput sequencing to reveal host phylogeny, diet, and environment as important host factors shaping mammalian gut microbiomes. Later studies further highlighted the role of host diet and phylogeny (Muegge *et al*., 2011; Groussin *et al*., 2017; Rojas *et al*., 2021) in shaping mammal gut microbiomes, however, the auto‐correlative nature between these two variables made them difficult to tease apart (Amato *et al*., 2018; Groussin, Mazel & Alm, 2020). Thus, uncovering generalizable ‘rules’ that govern animal gut microbiomes remains a difficult task.

Comparative gut microbiome research also revealed that results of comparative analyses are highly host dependent. For example, in primates, host phylogeny has a greater effect than host diet (Comizzoli *et al*., 2021; Gibson *et al*., 2019; Kinross *et al*., 2011; Worsley *et al*., 2021) in explaining gut microbiome diversity but the opposite is true for other mammals (Muegge *et al*., 2011; Youngblut *et al*., 2019). In fish, diet largely shapes surgeonfish (Acanthuridae) gut microbiomes (Miyake, Ngugi & Stingl, 2015) while the environment outweighs diet in a large number of fish species from the South China Sea (Kim *et al*., 2021). At a broader scale, phylosymbiosis (the degree to which gut microbiome compositions are specific to their host) is putatively higher in mammals than other vertebrate classes and also varies greatly across mammalian orders (Youngblut *et al*., 2019). By contrast, flight adaptation, rather than phylosymbiosis, may have determined gut microbiome composition in birds and bats (Song *et al*., 2020). These contrasting findings are pervasive across the animal kingdom, highlighting a potential phylogenetic dependency of how host factors determine gut microbiome composition and diversity and a need for more clade‐specific data.

Despite such contradictions, most comparative studies do show that gut microbiomes are highly multivariable – influenced by many host factors, such as diet (Muegge *et al*., 2011), gut anatomy (Amato *et al*., 2018), immune factors (Shi *et al*., 2017), and social behaviour (Song *et al*., 2013). What is not so obvious is how the effects of these host factors change in magnitude across different clades of hosts. Moreover, the extent to how microbes shape animal health and survival is unclear, particularly in non‐mammalian clades. For example, in humans and mice, *Akkermansia muciniphila* relative abundance is positively associated with high‐fibre diets and exercise and negatively associated with inflammation and disease (Geerlings *et al*., 2018; Naito, Uchiyama & Takagi, 2018). *Fecalibacterium prausnitzii*, another human‐associated microbe, has been implicated in a number of physiological processes involved in human health and disease (Cao, Shen & Ran, 2014; Lopez‐Siles *et al*., 2017; Parsaei *et al*., 2021) However, it is unclear whether such associations extend to other animal clades, including non‐mammals. Comparing host–microbe associations found in humans to other animal hosts can help us build a more accurate host–microbe model to help uncover the possible origins of these vital symbiotic relationships.

## II. MAMMALIAN GUT MICROBIOME DATA MAY BIAS GUT MICROBIOME THEORY

**Fig. 1:**
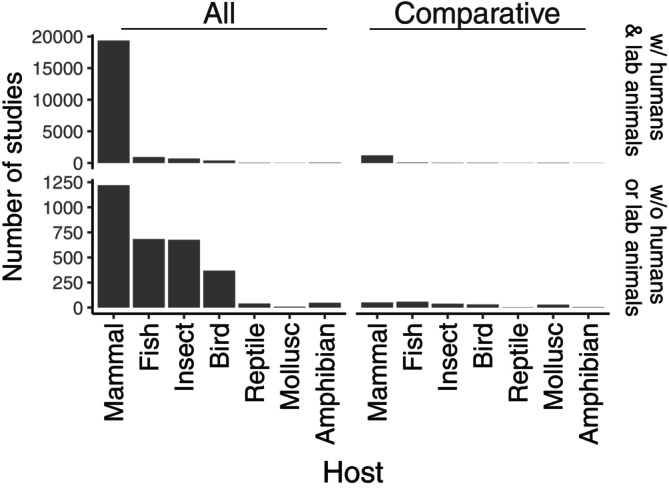
Number of gut microbiome studies across different host clades, and whether a study was comparative or not. Studies were collected in September 2023 using the *Scopus* document search tool. We counted only published journal articles and excluded all other article types (including reviews). The top row includes studies with human subjects and laboratory animals (w/ humans & lab animals) while the bottom row does not include these studies (w/o humans or lab animals). Both 16S and metagenomic studies are included.

Mammals are disproportionately studied in the gut microbiome literature relative to other clades (Fig. 1). When considering the diversity of clades such as fish (~29,000 species), reptiles (~12,000), birds (~10,000 species), and amphibians (~8000 species), compared to mammals (~5000 species), it is clear that the gut microbiome of the animal kingdom has not been sampled evenly (Colston & Jackson, 2016). Thus, most gut microbiome theory developed to date is based largely on mammal‐focused studies, potentially biasing the framework we use to construct models that attempt to explain the origins and functions of the gut microbiome. Moreover, a large majority of these studies are on humans and laboratory animals (compare upper and lower panels of Fig. 1). When considering only non‐comparative (i.e. single‐species) studies that sampled wild hosts, the numbers of studies between mammals and other taxa are more proportionate. There is thus reasonable taxon coverage in the existing literature and there is potential to analyse such data in a large‐scale comparative approach [see Hoffbeck *et al*. (2023) and Yang *et al*. (2022)]. Despite the availability of non‐mammalian gut microbiome studies, those that are highly cited tend to include only mammal‐centric data sets (Ley *et al*., 2008; Muegge *et al*., 2011; Amato *et al*., 2018; Youngblut *et al*., 2019), creating a bias may have influenced our efforts to understand the animal gut microbiome.

**Fig. 2:**
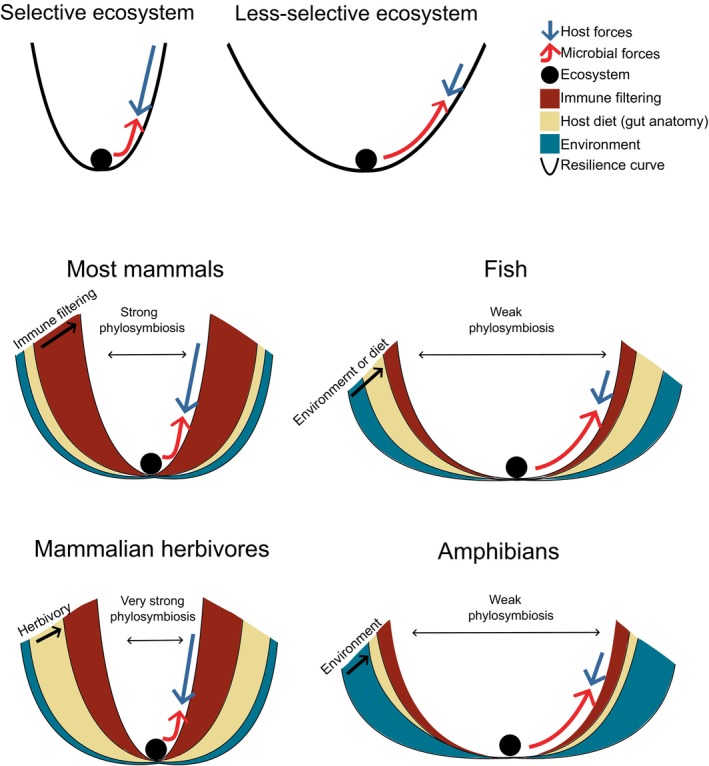
An ecosystem selection ‘ball and cup’ model of the animal gut microbiome that accounts for the multivariate shaping of gut microbiome composition across different host clades. A narrow resilience curve (black line on top left) results in a selective ecosystem due to strong host selective pressures such as immune filtering (blue arrow), whereas a wider curve (top right) results in a less‐selective ecosystem due to weaker host selective pressures and stronger microbial forces. The red arrow denotes stochastic microbial forces such as dispersal, genetic drift, and immigration. Each shaded colour under the resilience curve denotes a host factor that contributes to the width of the curve. We provide four example curves with varying widths across four groups of hosts. The width of the resilience curves is a proxy for the strength of phylosymbiosis within a given clade of hosts. The model predicts that tighter curves limit microbial forces due to highly selective host forces while wider curves allow the opposite to occur due to less‐selective host forces.

One example of such bias is in investigation of the importance of diet in shaping gut microbiome diversity. Host diet is often found to be an important driver of gut microbiome diversity in animal hosts. However, many studies that identified a large diet effect, did so with mammalian data sets (Ley *et al*., 2008; Muegge *et al*., 2011; Groussin *et al*., 2017; Youngblut *et al*., 2019). Moreover, significant effects of host diet have been repeatedly established in comparisons of mammalian carnivores and herbivores, which largely belong to two phylogenetically distinct orders (Carnivora and Artiodactyla), making it difficult to tease apart the effects of diet from those of host phylogeny. While the importance of host diet has been established in other animals (Hong *et al*., 2011; Miyake *et al*., 2015), there are also cases where no such effect was found [e.g. in primates (Amato *et al*., 2018) and the giant panda *Ailuropoda melanoleuca* (Wei, Wang & Wu, 2015; Guo *et al*., 2018)]. Thus, an overrepresentation of studies on carnivores and artiodactyls, which have very different gut anatomies and feeding adaptations (Muegge *et al*., 2011), may have skewed current views of the importance of diet in shaping gut microbiomes. It is more likely that the relative importance of different factors shaping animal gut microbiomes will vary greatly from clade to clade (Fig. 2). Diet may well be an important driver of gut microbiome diversity, however comparisons of a wider range of hosts with different dietary adaptations across the animal kingdom are needed.

## III. ESTABLISHING A COMPREHENSIVE GUT MICROBIOME THEORY

Various ecological theories have been applied to animal gut microbiomes. Currently, much gut microbiome ecological theory stems from human models (McDonald, Marchesi & Koskella, 2020; Wolff, Shoemaker & Garud, 2020), and unifying theories that account for hosts across the animal kingdom are lacking (Koskella, Hall & Metcalf, 2017). For example, many theoretical gut microbiome studies assume there is coevolution between gut microbes and their hosts (Zeng *et al*., 2015; Ma, 2021). Ma (2021, p. 1) states that ‘Animal (human) gut microbiomes have been coevolving with their hosts for many millions of years’. While coevolution of gut microbes and their animal hosts may indeed take place, the supporting evidence stems largely from work on hominid hosts (Moeller *et al*., 2014, 2016; Sanders *et al*., 2023). Recently, the concept of host–microbiome coevolution has been questioned (Groussin *et al*., 2020) and models that highlight ecology rather than evolution may describe better the variation within animal gut microbiomes (Colston & Jackson, 2016). In this section, we provide a summary of ecological models that have been applied to gut microbiomes and provide an updated framework that places these models in a more comprehensive light, using metacommunity theory. For clarity, we divide these models into top‐down host‐imposed forces and bottom‐up microbial forces.

A variety of ecological models have been proposed that focus on host factors that shape microbe community assembly. Here, we refer to these as top‐down host‐imposed forces, highlighting the host's ability to shape and facilitate gut microbiome community assembly. Habitat filtering and niche‐based selection have been discussed extensively as ecological frameworks that can be applied to host modulation of microbiomes (Weiher, Clarke & Keddy, 1998; Fierer *et al*., 2012; Costello *et al*., 2012). Habitat filtering (in contrast to niche differentiation) describes the process by which species with similar functional niches coexist in similar environments (Keddy, 1992; Díaz, Cabido & Casanoves, 1998; Yang *et al*., 2019). Habitat filtering can explain microbiome variation across different habitats within a given host. For example, even after controlling for phylogeny, coexisting microbes are more likely to share similar metabolic functions within the same environment (Levy & Borenstein, 2013). In the context of gut microbiomes, the host can be viewed as the habitat or environment, where various parameters such as pH, temperature, and immune function can select or filter for certain microbes. As a result, the gut microbiome is a very different community compared to microbial communities on other body sites (e.g. the skin microbiome) (Dekaboruah *et al*., 2020), and this differentiation has been documented across a wide variety of animal hosts (Grice & Segre, 2011; Sylvain *et al*., 2020; Degregori, Casey & Barber, 2021). However, gut microbiome composition and diversity can vary greatly between different host species (Youngblut *et al*., 2019; Song *et al*., 2020) and determining whether this variability is due to variation in top‐down host‐imposed forces requires further investigation.

While host‐oriented approaches can be used to model gut microbiome community composition and diversity well on broad scales, they do not account fully for the significant stochasticity observed in gut microbiome communities, particularly among individuals of the same host species. Neutral models assuming stochastic processes such as immigration (e.g. microbes colonising the gut), extinction, genetic drift, and speciation (Hubbell, 2001), have been proposed as possible frameworks to understand microbial community assembly within the gut from the perspective of bottom‐up microbial forces (Costello *et al*., 2012). However, while neutral models can accurately describe gut microbiome variability in theory (Zeng *et al*., 2015), they can fail when applied to actual gut microbiome data sets (Li & Ma, 2016). More deterministic processes such as niche partitioning (Yu *et al*., 2024), may also explain microbial variability in gut microbiomes, but efforts to test niche partitioning in gut microbiomes are lacking.

Specific microbe–microbe interactions such as cooperation, mutualism, and competition are also significant determinants of microbiome composition in the gut (Coyte & Rakoff‐Nahoum, 2019). For example, Segura Munoz *et al*. (2022) showed that *Akkermansia muciniphila* and *Bacteroides vulgatus* exhibited significantly different levels of competitive behaviour and species exclusion within the mouse gut. However, in zebrafish (*Danio rerio*), the effects of microbe–microbe interactions appear to diminish with increasing gut microbiome complexity (Sundarraman *et al*., 2020). Microbes can also exhibit cooperative and mutualistic behaviours within the gut, such as the cross‐feeding enzyme system between *Bacteroides vulgatus* and *B. ovatus* (Coyte & Rakoff‐Nahoum, 2019) or between *Lactobacillus plantarum* and various gut microbes (Heinken & Thiele, 2015). Such cooperative behaviours lead to a network of syntrophic relationships among microbial species within the gut (Culp & Goodman, 2023). Thus, microbe–microbe interactions may be as important as host‐imposed forces in controlling gut microbiome community assembly and diversity.

To account for both microbe and host‐imposed forces, we propose a metacommunity theory approach to animal gut microbiomes. Metacommunity theory attempts to consolidate niche‐based selection and neutral theory into a unifying framework (Fierer *et al*., 2012). Here, we take a similar but slightly modified approach that assumes that both top‐down selection imposed by the environment (i.e. the host) and bottom‐up microbial forces occurring within the microbial community work simultaneously to shape gut microbiome composition (Fig. 2). In our proposed model, these two opposing forces vary among different host clades. Gut microbes likely undergo varying levels of selection imposed by different host factors, leading to co‐diversification or phylosymbiosis. Because mammals putatively impose the strongest selection on their gut microbiomes (Mallott & Amato, 2021), mammalian gut microbiomes may particularly exhibit patterns that mimic host–microbe coevolution in contrast to other hosts. Simultaneously, stochastic microbial forces, such as dispersal, horizontal gene transfer, and rapid evolution, counteract host selection, and may dominate the shaping of gut microbiomes in hosts with weaker selection. Building on this framework, future studies should investigate whether stochastic microbial forces are more pronounced in non‐mammalian hosts with weaker host selection on their gut microbiomes and whether the opposite is true in mammalian hosts.

## IV. USE OF HOST PHYLOGENY AS AN ALL‐ENCOMPASSING FACTOR

Host phylogeny, sometimes used interchangeably with phylosymbiosis, can conveniently help researchers capture the complexity of host species differences into a single variable for analysis. However, finding consistent patterns between animal gut microbiome diversity and host phylogeny has proved difficult (Mallott & Amato, 2021). For example, among vertebrates, mammals have the highest levels of phylosymbiosis, with artiodactyls showing the highest within‐taxon gut microbiome similarity (Song *et al*., 2020), but the extent to which this is confounded by their herbivorous diet remains unclear (Muegge *et al*., 2011). Host phylogeny has been shown partly to shape fish gut microbiomes (Sullam *et al*., 2012), but contradictory examples exist (Givens *et al*., 2015; Miyake *et al*., 2015; Kim *et al*., 2021). It has been proposed that mammal gut microbiomes may exhibit the strongest phylosymbiosis as a result of their complex immune systems (Woodhams *et al*., 2020; Mallott & Amato, 2021), and host phylogeny can greatly outweigh host ecology in shaping gut microbiome diversity in different mammalian clades (Wei *et al*., 2015; Amato *et al*., 2018). More diverse sampling could help us understand why phylosymbiosis appears to be strongest in mammals compared to other animal clades.

Furthermore, while terms such as ‘phylosymbiosis’ and ‘host phylogeny’, can be useful in describing gut microbiome variation across different hosts, it can be misleading in terms of how gut microbiomes fit into animal ecology and evolution. Comparative studies often separate potential host factors for analysis into broad categories such as host phylogeny, host diet, and host environment (Ley *et al*., 2008; Sullam *et al*., 2012; Groussin *et al*., 2017; Youngblut *et al*., 2019). However, they may be intercorrelated: host diet, for example, is an evolutionary trait that is correlated with phylogeny (Román‐Palacios, Scholl & Wiens, 2019). Thus, a study claiming a greater effect of host diet than of host phylogeny is actually arguing that one defined evolutionary trait (diet) is outweighing all other evolutionary traits of that host (phylogeny). In physiological terms, host diet outweighing host phylogeny can be seen as digestive anatomy outweighing the sum of all non‐dietary physiology such as immune function, thermoregulation, liver metabolism, and many other physiological functions. Delsuc *et al*. (2014), for example found the effects of insectivory outweighed host phylogeny in determining the microbiome of certain myrmecophagous mammals. Because insectivory evolved independently multiple times in mammals, convergence of their gut microbiome highlights the impact of their recent dietary evolution over their other biological traits. By contrast, Amato *et al*. (2018) found that the effects of host phylogeny outweighed those of host diet in the gut microbiome of primates, and attributed the correlation with host phylogeny to host physiology. While host phylogeny can serve as a useful proxy, more comprehensive physiological sampling of the host could help researchers pinpoint specific physiological variables that drive gut microbiome variability.

## V. ENVIRONMENTAL IMPACTS VARY ACROSS HOST CLADES

Different biomes have very distinct microbial compositions (Thompson *et al*., 2017), and it is thus possible that gut microbiomes reflect this diversity. However, given the differences in temperature, pH, nutrient levels, and oxygen content in the gut compared to the external environment, it is also possible that gut microbiomes may not reflect the external environment at all. Many contradictory results exist in the literature showing the environment to have either a large or minimal role in shaping animal gut microbiomes. For example, Kim *et al*. (2021) found that saltwater and freshwater fish from the South China Sea region exhibited distinct gut microbiome compositions. The effect of host environment outweighed other investigated factors such as host phylogeny and diet. Similar findings have been reported for reptiles (Hoffbeck *et al*., 2023; Moeller *et al*., 2020; Vasconcelos *et al*., 2023) and amphibians (Bletz *et al*., 2016). By contrast, comparative studies on mammals often find little effect of environment (Youngblut *et al*., 2019; Song *et al*., 2020). Some environment‐related effects have been identified in single‐species studies on mammals (Song *et al*., 2013; Degregori *et al*., 2023) or in comparisons of closely related species (Grieneisen *et al*., 2019), perhaps due to isolation of the effects of the environment from phylogeny. It appears that environmental impacts on animal gut microbiomes are stronger in clades with lower levels of phylosymbiosis such as fish, reptiles, and amphibians (Fig. 2), while in mammals, any effects of host environment may be masked by other factors such as host physiology. Additional comparative research is needed to confirm this hypothesis.

Multiple challenges exist that limit our ability to isolate the effects of environment on animal gut microbiome diversity and function. Obtaining wild samples, for example, can be difficult and costly, and so researchers often resort to sampling captive hosts (Ley *et al*., 2008; Hale *et al*., 2018; Gibson *et al*., 2019), whose gut microbiomes are unlikely to be representative of their wild counterparts (Clayton *et al*., 2016; McKenzie *et al*., 2017; Alberdi, Martin Bideguren & Aizpurua, 2021) and may reflect the microbiome of the captivity environment (Clayton *et al*., 2016; Dehler, Secombes & Martin, 2017). When researchers can access wild hosts, the challenge of an effective study design arises, where sufficient variation in host habitats must be sampled to analyse properly the effect of host environment. Moreover, to isolate an effect of environment from that of other factors such as host phylogeny and ecology, an effective study design must include not only a variety of habitats, but also a variety of host species with varying biological and ecological traits.

## VI. DETERMINING SAMPLE SIZE IN COMPARATIVE GUT MICROBIOME RESEARCH

In addition to the challenges of sampling diverse environments, comparative gut microbiome studies also face the costs associated with sample collection and sequencing. One recent estimate found the average cost of a 16S sequencing workflow to be around $29 per sample (Lao, 2022), if a developed laboratory pipeline is in place. For new laboratories, equipment and field collection costs could increase this to in excess of $50–100 per sample. Thus, comparative microbiome studies often face a challenging trade‐off due to budget limitations: choosing either a large number of host species with a low number of replicates, or more replicates with fewer host species. In addition, targeting wild hosts over captive hosts can incur significantly higher costs, require additional expertise, and involve travel to multiple remote locations to collect samples with approved permits. Thus, it may be the case that a comparative gut microbiome study is not possible within a given budget.

**Fig. 3:**
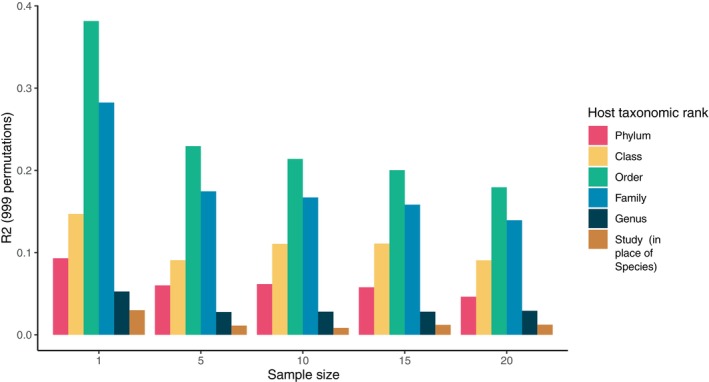
Results of Adonis analysis of the effects of sample size per host species on the calculated influence of taxonomic rank on animal gut microbiomes. Samples from each host were randomly subsampled at 999 permutations; the R2 value plotted is the mean from all permutations. Error bars are too small to show on the graph but were all <0.001. Colours denote host taxonomic ranks which were treated as separate factors. Because Study and Species are nearly identical (most studies had a unique host species), study, as a factor, is included to explore the batch effect after taking taxonomic rank into account. We rarefied the data to 1000 reads and used unweighted UniFrac distance matrices as inputs for the Adonis test.

To investigate how many replicate samples per host species are needed, we aggregated data from 179 gut microbiome studies (see online Supporting Information, Appendix S1, for methods; Table S1 for search results, Data S1 for the list of included studies, and Fig. S1 for sample counts across host clades) and randomly pooled these into data sets containing different numbers of samples sizes per host species (Fig. 3). We found that the influence of phylogeny was much larger for low sample sizes, with the largest effect at a sample size of one, and a more consistent effect after about five replicate samples per host species. A batch effect also may be present due to combining studies (‘study’ in Fig. 3), but we instead focus on how the results change across sample sizes rather than the actual effect sizes for each taxonomic rank.

Gut microbiome studies often utilise public data sets from other comparative studies in phylogenetic comparisons with their study species (Delsuc *et al*., 2014; Youngblut *et al*., 2019; Song *et al*., 2020; Sanders *et al*., 2023). Such comparisons can enable researchers to identify convergences between distantly related hosts (Muegge *et al*., 2011; Delsuc *et al*., 2014; Song *et al*., 2020; Degregori *et al*. 2024) or to identify elements of the gut microbiome that follow host evolution (Moeller *et al*., 2014; Groussin *et al*., 2017). However, the majority of comparative studies have been focused on mammals, and comprehensive data sets of other clades are lacking (Table S2). While such data sets are emerging, such as for fish and birds (Song *et al*., 2020; Kim *et al*., 2021; Minich *et al*., 2022), data sets that span the entire animal kingdom are not yet available (although see Yang *et al*., 2022). An easily filterable comparative data set of gut microbiomes across the animal kingdom would provide researchers with the ability to conduct robust evolutionary and ecological analyses.

**Fig. 4:**
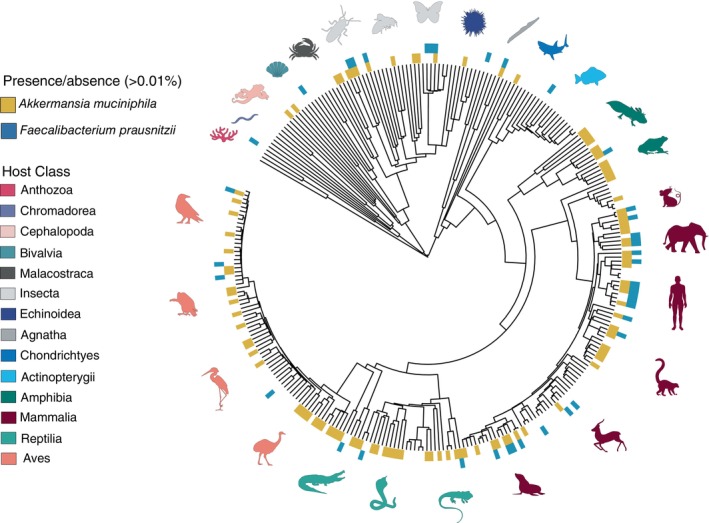
An annotated phylogeny of the presence of *Akkermansia muciniphila* and *Faecalibacterium prausnitzii* in the gut microbiomes of hosts from 179 compiled studies. Orange and blue colours at the tips denote the presence of these gut microbes. Each tree tip represents a host species included in our database.

As shown in Fig. 1, when excluding studies on humans and laboratory animals, there is a reasonably comparable number of single‐species studies, across various vertebrate and invertebrate clades. Combining results from single‐species studies to curate a comparative data set can serve as a low‐cost alternative approach for researchers compared with the high costs of generating an original comparative data set. While prone to individual laboratory processing biases, studies can be combined on the basis of using identical primers such as the commonly used V3‐V4 (341F‐806R) and V4 (515F‐806R) primers to minimise such bias. To show the analytical power of such an approach, we used our database of 179 gut microbiome studies (see Data S1) to examine the prevalence of two commonly studied microbes, *Akkermansia muciniphila and Faecalibacterium prausnitzii*, across the animal kingdom (Fig. 4). While both microbes are of interest in human biomedical studies (Naito *et al*., 2018; Parsaei *et al*., 2021), Fig. 4 shows that, among vertebrates, *A. muciniphila* appears to be widespread across vertebrate gut microbiomes while *F. prausnitzii* is present most often in mammalian hosts. This type of broad‐scale analysis could guide researchers to focus their efforts on particular host species of interest and their closest relatives.

## VII. BEST PRACTICES AND FUTURE DIRECTIONS

Comparative analyses of the gut microbiome can utilise 16S data, metabolomic data, transcriptomic data, metagenomic data, or even proteomic data (Knight *et al*., 2018). These data can then be compared against a range of host variables, including ecological data, host phylogeny, biometric data or physiological measurements (Gomez *et al*., 2015; Amato *et al*., 2018; Youngblut *et al*., 2019). Thus, comparative gut microbiome studies vary greatly in their methodologies and study designs.

Moving forward, general guidelines are needed for future comparative gut microbiome research to improve standardisation. While any comparative study has its own specific goals and limitations, standardising methodology and study design could greatly improve our ability to investigate more general aspects of how gut microbiomes fit into animal ecology and evolution. Without such standardisation we are forced to limit any conclusions to specific studies and the specific methodology they employed. We therefore provide the following recommended guidelines for future comparative work.

### (1) Include publicly available data in comparative studies

The increasing availability of public gut microbiome data from hosts across the animal kingdom provides an opportunity for researchers to perform comparative analyses of the gut microbiomes of their target host(s). For example, studies that only compare two host species, such as yaks and pikas (Fu *et al*., 2020) or two different amphipods (Cheng *et al*., 2019), could utilise such supplementary data to investigate whether differences in gut microbiomes between two hosts are driven by host phylogeny, diet, or environment. As noted in Section VI, studies using publicly available data will have to consider primer bias, and ideally focus on results from studies with the same primer (Darwish *et al*., 2021).

### (2) Collect a minimum of five samples per host species

Comparative gut microbiome studies vary widely in how many host species are targeted and how many samples are collected per host. Some studies include many host species but only collect one sample per host (Ley *et al*., 2008; Levin *et al*., 2021), while at the other extreme a study may obtain many samples per host for only one or a few host species (Zhang, Ren & Gong, 2019; Vasconcelos *et al*., 2023). Combining the results of such studies can be difficult. Moreover, as shown in Fig. 3, undersampling could lead to overestimations of impacts of host phylogeny on animal gut microbiomes. We recommend sampling a minimum of five samples per host species. Additional samples should be allocated towards further host diversity as we show that potential source of bias is lower for sample sizes of five or greater. However, in some cases this minimum may be insufficient. Comparative analyses focused on a specific variable should perform a power analysis to ensure an adequate sample size (Rahman *et al*., 2023). There is a lack of statistical tools to help scientists determine the appropriate adequate amount of host diversity for comparative analyses. While we suggest that a sample size of 5 per host species is adequate for some analyses, such as Adonis, further tools to determine the appropriate sampling level at higher taxonomic ranks, such as host family or phylum, or in studies using specialised statistical models, are needed.

### (3) Accurately quantify diet

Comparative gut microbiome studies often group hosts into broad diet categories for analysis (Ley *et al*., 2008; Muegge *et al*., 2011; Groussin *et al*., 2017). However, more accurate measures of diet composition, such as diet metabarcoding (Kartzinel *et al*., 2019) or survey data (Song *et al*., 2020), would allow researchers to analyse better how diet impacts animal gut microbiomes across host species. Moreover, categories such as ‘herbivory’ and ‘carnivory’ in mammals, for example, may be significantly correlated with host phylogeny (Ley *et al*., 2008; Groussin *et al*., 2020) and may also differ in meaning depending on the host clade of interest. Fish and mammal carnivores, for example, may differ greatly in their respective feeding strategies (Román‐Palacios *et al*., 2019), but such differences are ignored by coarse diet categories. We recommend that comparative studies should use quantifiable measures of host diet instead of broad categories.

### (4) Collect host physiological data

In humans and mouse models, gut microbiomes are significantly associated with many aspects of host physiology (Besten *et al*., 2013; Barouei *et al*., 2017; Thiele *et al*., 2020), but the extent to which this is true in other taxa remains unclear. For instance, it has been posited that immune factors may play a large role in shaping gut microbiome composition across vertebrates (Woodhams *et al*., 2020; Mallott & Amato, 2021), but comparative approaches that also sample host physiology, such as red or white blood cell counts, across a diverse set of host species are lacking. Sampling a range of physiological variables, such as the host metabolome, immune factors, or levels of cortisol and testosterone, could provide a more complete picture of how animal physiology shapes the gut microbiome and how this varies across the animal kingdom. Authors then will be able to make stronger inferences on the differences underlying gut microbiome diversity among species instead of resorting to host phylogeny as the underlying mechanism.

### (5) Moving beyond host phylogeny as a host factor

Comparative studies often cite host phylogeny as a factor determining differences in gut microbiomes among animal species. As described previously, host phylogeny can serve as a useful tool to investigate how differences in gut microbiomes correlate with host evolution. However, treating host phylogeny, host diet, and host environment as separate factors can be problematic considering that host diet also is an evolved trait and all animals will have evolved adaptations to their environment. We encourage researchers, when possible, to collect physiological data to supplement the use of host phylogeny in analyses of how gut microbiomes are correlated with host evolution.

## VIII. CONCLUSIONS

(1) Comparative work on animal gut microbiomes has shown that ecological and evolutionary factors can shape gut microbial communities, and that these effects are host dependent and vary among taxa. The underlying mechanisms are poorly understood due to the lack of comprehensive meta‐analyses on comparative data sets, including data sets with non‐mammalian hosts. Our proposed ecosystem‐selection framework accounts for the stronger phylosymbiosis and larger core microbiome we see in mammals while predicting weaker phylosymbiosis in other clades.
(2) To test this hypothesis and other predictions, data sets that are rich in host diversity and span the entire animal kingdom are needed. Generating such data with standardised field and laboratory practices will be critical and quantifying host physiology in addition to host phylogeny can help us delineate the complex network of host factors that shape gut microbiome diversity.
(3) We show that the aggregation of individual studies into large‐scale data sets is possible and can be used to investigate broad questions concerning the relationship between gut microbiomes and animal ecology and evolution. Future efforts should seek to combine 16S, metagenomic, and metabolomic data sets, with rich host biological and ecological data, to enable a more detailed understanding of both the diversity and function of animal gut microbiomes.

## AUTHOR CONTRIBUTIONS

S. D. and K. R. A. conceived the study. X. W. led data collection and curation. A. K., N. S., S. M., A. M., K. E., S. J., E. S., E. K., K. S., Z. H., S. D., R. K., and K. R. A aided in data collection. S. D., N. S., A. K., and A. M. developed code for analyses. S. D. carried out analyses and manuscript writing. K. R. A. and R. K. provided computational resources and guidance. All authors provided feedback on the analyses and editing of the manuscript.

## Supporting information


**Appendix S1.** Supplementary methods.
**Table S1.** Web of Science query results.
**Table S2.** Overview of large‐scale comparative gut microbiome studies (>30 host species).
**Fig. S1.** Number of samples aggregated across 179 gut microbiome studies spanning 15 host classes.

**Data S1.** Sample and study metadata.

## Data Availability

All metadata used in this study along with a list of compiled studies are available in the supporting information and at https://github.com/samd1993/GutMicrobiomeTreeOfLife as an excel spreadsheet named: ‘Degregori_etal_Comparative_Review_metadata_Oct20_24.xlsx’ and a text file of related scripts named: ‘Degregori_etal_Comparative_Review_DataScripts.txt’. The sequence table used for Figs 3 and 4 is named ‘N30GMTOLsolo_table.biom’ and is also uploaded to GitHub. The package q2sra is available at https://pypi.org/project/q2sra/.
