# Supplementary material for: Comparative gut microbiome research through the lens of ecology: theoretical considerations and best practices
Source: Biol Rev Camb Philos Soc. 2024 Nov 12;100(2):748–63. doi: 10.1111/brv.13161 (PMC11885713; doi:10.1111/brv.13161)
Supplement: Supplementary file 1 — Appendix S1. Supplementary methods. Table S1. Web of Science query results. Table S2. Overview of large‐scale comparative gut microbiome studies (>30 host species). Fig. S1. Number of samples aggregated across 179 gut microbiome studies spanning 15 host classes. [file BRV-100-748-s001.docx]

**Appendix S1. Supplementary Methods**

*Data aggregation*

To build a representative comparative data set of gut microbiota across the animal kingdom, we used *Web of Science* to search for 16S gut microbiome studies on animals spanning the animal kingdom, and *Google Scholar* to search for this information in certain taxa (e.g. annelids, lampreys, and echinoderms) for which we found no studies in *Web of Science*. We did not include years in our search queries since 16S high throughput sequencing studies only appear after 2006, with a majority that are actually usable (accessible data and metadata) occurring after 2012. Our search string and results are provided in Table S1. We included studies that used either V3-V4 or V4 primers and excluded studies without metadata. To aggregate information from the selected studies, we downloaded saw gut microbiome sequence data from the NCBI database, and wrote an automated script to process the sequence data and collect study metadata, which included host species taxonomy (species, genus, family, order, class, phylum), number of samples collected, study accession number, and the study’s first author name. We have made this script publicly available as a python package titled, “q2sra”. Script source code and functionality can be accessed at: https://github.com/noahschulhof/q2sra. This package can cross-reference whether the studies are accessible on a public repository. Thus, this step serves as an additional filter we used after our search query filters, to exclude studies that did not make their 16S gut microbiome data publicly accessible with usable metadata. This left us with roughly 200 studies with metadata that was also verified manually by all the undergraduate research assistants who are listed as authors in this study and who were also involved in study collection. We collected a maximum of 30 gut microbiome samples per study; in total 3,900 samples were collected. We capped the number at 30 for data storage reasons. However, some studies included less than 30 total samples. Studies with less than 20 samples were not used in the sample size analysis but were used in other diversity analyses. We refrained from using studies of hosts in captivity unless a study on captive animals was the only one available for that host. We did not include studies of juveniles, ill animals, or animals given medical treatments, particularly antibiotics. Data S1 (also available at: https://github.com/samd1993/GutMicrobiomeTreeOfLife/blob/main/Degregori_etal_Comparative_Review_metadata_Oct20_24.xlsx) provides metadata for each study, with host accession numbers. We aggregated information from 179 studies spanning 15 host classes, of which seven were invertebrates. Six studies included two different host species, leading to a total of 185 hosts from 179 different studies, and 163 unique host species.

*Data processing*

To process the aggregated data, we ran deblur on the forward reads, trimming at 150bp, and then merged all reads into a single count table and built a tree using MAFFT. Both deblur and MAFFT were used within the QIIME2 v2023.2 platform (Bolyen *et al*., 2019). We only used the forward reads because a majority of studies shared the same starting forward position, either at 515F or 341F of the 16S gene in contrast to the reverse reads which varied greatly in starting position. To assign taxonomy we used a Greengenes 2 (McDonald *et al*., 2023) classifier trained on the entire 16S region to ensure both V3 and V4 regions were classified properly. Any sequence assigned as a eukaryote or as mitochondrial DNA was removed from the data set. Any chloroplast not pertaining to cyanobacteria was also removed. The taxonomy file is uploaded to our github as a qiime2 qza file (See Data Availability for further details).

*Data analysis*. For the Adonis (PERMANOVA) tests of the effects of using different sample sizes, we created randomly generated data sets with either 1, 5, 10, 15, or 20 samples per host through random subsampling. We performed this subsampling for 999 permutations to minimize sampling bias. For the Adonis test we rarefied the data to 1000 reads which allowed us to retain 90% of the samples, and used UniFrac distance matrices as inputs. Our Adonis formula was: UniFrac distance ~ Phylum + Class + Order + Family + Genus + Study. Because almost every species was its own study, Study and Species were essentially the same and so we use Study as the last factor to explore the batch effect after taking into account other phylogenetic ranks.

We did not rarefy sequence data to perform the *A. muciniphila* and *F. prausnitzii* diversity surveys since we used relative abundance as a cutoff measure (>0.01%) instead of absolute sequence counts. Samples were pooled and the median relative abundance used *in lieu* of the sum relative abundance to avoid single samples having a disproportionate effect on the pooled relative abundance.

**Table S1.** *Web of Science* query results. Queries (in March 2023) took the form of "<host clade> gut microbiome" for each clade and excluded humans. We then excluded non-relevant studies by adding subsequent queries to the initial query in the following order: 16S, V3-V4 OR V4, excluding lab experiments AND antibiotics. Finally, we used q2sra to exclude studies without metadata or identifiable sample IDs which are necessary for downstream processing and analyses. We specified Research Articles Only. Three studies included two different host species.

| Initial query | **Host** | **Article count** |
| --- | --- | --- |
|  | Mammal | 448 |
|  | Reptile | 39 |
|  | Bird | 368 |
|  | Fish | 919 |
|  | Insect | 675 |
|  | Mollusc | 27 |
|  | Amphibian | 135 |
|  | Total | **2611** |
| Including 16S | Mammal | 361 |
|  | Reptile | 34 |
|  | Bird | 298 |
|  | Fish | 679 |
|  | Insect | 481 |
|  | Mollusc | 23 |
|  | Amphibian | 110 |
|  | Total | **1986** |
| Including V3-V4 or V4 | Mammal | 302 |
|  | Reptile | 29 |
|  | Bird | 164 |
|  | Fish | 371 |
|  | Insect | 334 |
|  | Mollusc | 18 |
|  | Amphibian | 65 |
|  | Total | **1283** |
| Excluding lab animals | Mammal | 133 |
|  | Reptile | 27 |
|  | Bird | 87 |
|  | Fish | 107 |
|  | Insect | 92 |
|  | Mollusc | 17 |
|  | Amphibian | 39 |
|  | Total | **502** |
| Excluding studies without metadata | Mammal | 32 |
|  | Reptile | 20 |
|  | Bird | 7 |
|  | Fish | 24 |
|  | Insect | 50 |
|  | Mollusc | 15 |
|  | Amphibian | 11 |
|  | Other* | 20 |
|  | Total | **179**^†^ |
| *****Manual searches in *Google Scholar* for other taxa (annelids, echinoderms, lampreys, Holothuroidea, and a set of human studies covering multiple geographies around the globe).  ^†^6 studies contained 2 different host species leading to a total of 185 hosts overall, and 22 studies targeted the same host (11 targeted humans) leading to 163 unique host species in our database. | | |

**Table S2.** Overview of large-scale comparative gut microbiome studies (>30 host species).

| **Study** | **Host** | **Sample size** | **no. of species** | **# of samples per species** | **# of host classes** | **year** |
| --- | --- | --- | --- | --- | --- | --- |
| Muegge *et al*. (2011) | Mammals | 38 | 33 | 1 | 1 | 2011 |
| Jonge *et al*. (2022) | Mammals | 66 | 52 | 1 | 1 | 2022 |
| Youngblut *et al*. (2019) | Vertebrates | 213 | 128 | 2 | 4 | 2019 |
| Milani *et al*. (2020) | Mammals | 250 | 77 | 3 | 1 | 2020 |
| Kim *et al*. (2021) | Fish | 227 | 85 | 3 | 1 | 2021 |
| Hird *et al*. (2015) | Birds | 129 | 46 | 3 | 1 | 2015 |
| Minich *et al*. (2022) | Fish | 416 | 101 | 4 | 1 | 2022 |
| Song *et al*. (2020) | Vertebrates | 6141 | 987 | 5 | 4 | 2020 |
| Hoffbeck *et al*. (2023) | Reptiles | 745 | 91 | 7 | 1 | 2023 |
| Our data set | Vertebrates | 4639 | 191 | 30 | 19 | 2016–23 |

**
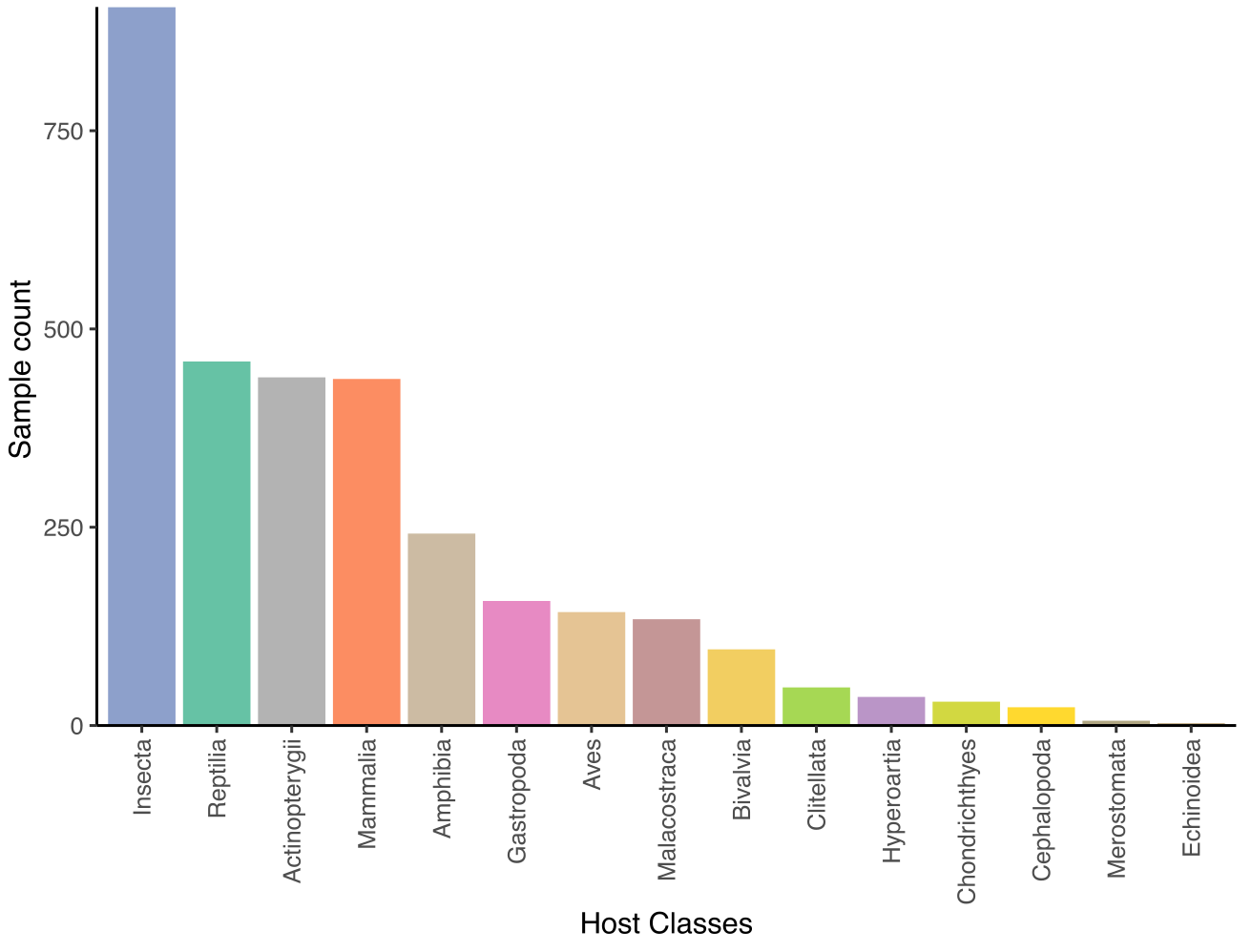
**

**Fig. S1.** Number of samples aggregated across 179 gut microbiome studies spanning 15 host classes.
